# Replication of HLA class II locus association with susceptibility to podoconiosis in three Ethiopian ethnic groups

**DOI:** 10.1038/s41598-021-81836-x

**Published:** 2021-02-08

**Authors:** Tewodros Gebresilase, Chris Finan, Daniel Suveges, Tesfaye Sisay Tessema, Abraham Aseffa, Gail Davey, Konstantinos Hatzikotoulas, Eleftheria Zeggini, Melanie J. Newport, Fasil Tekola-Ayele

**Affiliations:** 1 Armauer Hansen Research Institute (AHRI), Addis Ababa, Ethiopia; 2 Unit of Health Biotechnology, Institute of Biotechnology, College of Natural and Computational Sciences, Addis Ababa University, Addis Ababa, Ethiopia; 3 Institute of Cardiovascular Science, Faculty of Population Health, University College London, London, UK; 4 Wellcome Trust Sanger Institute, Hinxton, Cambridge, UK; 5 Brighton and Sussex Centre for Global Health Research, Brighton and Sussex Medical School, Brighton, UK; 6 Epidemiology Branch, Division of Intramural Population Health Research, Eunice Kennedy Shriver National Institute of Child Health and Human Development, National Institutes of Health, Bethesda, MD USA; 7 Present Address: European Bioinformatics Institute, Hinxton, Cambridge, UK; 8 Present Address: Institute of Translational Genomics, Helmholtz Zentrum München – German Research Center for Environmental Health, Neuherberg, Germany; 9 Present Address: TUM School of Medicine, Technical University of Munich and Klinikum Rechts Der Isar, Munich, Germany

**Keywords:** Immunopathogenesis, Genetic association study, Immunogenetics, Genetics, Pathogenesis, Medical research, Genetics research, Diseases, Immunological disorders, Skin diseases

## Abstract

Podoconiosis, a debilitating lymphoedema of the leg, results from barefoot exposure to volcanic clay soil in genetically susceptible individuals. A previous genome-wide association study (GWAS) conducted in the Wolaita ethnic group from Ethiopia showed association between single nucleotide polymorphisms (SNPs) in the HLA class II region and podoconiosis. We aimed to conduct a second GWAS in a new sample (N = 1892) collected from the Wolaita and two other Ethiopian populations, the Amhara and the Oromo, also affected by podoconiosis. Fourteen SNPs in the HLA class II region showed significant genome-wide association (*P* < 5.0 × 10^−8^) with podoconiosis. The lead SNP was rs9270911 (*P* = 5.51 × 10^−10^; OR 1.53; 95% CI 1.34–1.74), located near *HLA-DRB1*. Inclusion of data from the first GWAS (combined N = 2289) identified 47 SNPs in the class II HLA region that were significantly associated with podoconiosis (lead SNP also rs9270911 (*P* = 2.25 × 10^−12^). No new loci outside of the HLA class II region were identified in this more highly-powered second GWAS. Our findings confirm the HLA class II association with podoconiosis suggesting HLA-mediated abnormal induction and regulation of immune responses may have a direct role in its pathogenesis.

## Introduction

The neglected tropical disease podoconiosis is a type of progressive tropical lymphoedema that mainly affects the lower leg. It results from long term exposure of bare feet to red clay soil derived from volcanic rock^1^. The disease has been described in at least 32 countries, affecting susceptible individuals who do not wear shoes consistently^2,3^. It is estimated that there are 4 million people living with podoconiosis globally, mainly living in highland regions of tropical countries in Africa, South and Central America and southeast Asia^3^. Ethiopia bears the largest burden of cases where nationwide disease mapping indicated a prevalence of 4%^4^ amounting to 1.5 million adults living with podoconiosis and a further 34.9 million at risk of the disease^5^. In addition to its physical consequences, which include disability and painful inflammatory episodes affecting the lower limb (known as acute dermatolymphangioadenitis)^6^, podoconiosis has considerable negative economic^7^ and psychosocial impact^8–10^ on affected individuals, families and communities.

Little is known about the pathogenesis of the disease. Early histopathology and electron microscopy studies suggest that soil particles cross the skin, are taken up by macrophages and transported to regional lymph nodes^11^. It was proposed that the immune system is activated leading to inflammation and scarring particularly affecting the lymphatic system. The lymph vessels gradually become obstructed causing progressive lymphoedema with corresponding skin changes in the lower limb that are typical of podoconiosis. These include dermal nodules and a rough, velvet-like appearance to the skin known as mossy changes which are pathognomonic for the disease.

The precise nature of the soil trigger is unknown but specific geological and climate conditions are required that lead to the formation of irritant soil, which explains the geographical distribution of podoconiosis in volcanic highland areas^12^. Differences in particle and mineral composition between soil from endemic and non-endemic regions have been described, but correlation with the ability of the different soils to induce inflammation measured by haemolytic activity was not demonstrated^13^.

There is also evidence that genetic factors play a role in the pathogenesis of podoconiosis. This was first provided by a study conducted in Ethiopia in 1972, which demonstrated familial clustering of cases^14^. A segregation analysis of 59 multi-generational podoconiosis families from the Wolaita ethnic group in southwest Ethiopia indicated an autosomal co-dominant pattern of inheritance with an estimated sibling recurrence risk ratio (λs) and heritability of 5.07 and 0.63 respectively^15^. A genome-wide association study (GWAS) of 194 cases and 203 controls from the same population identified genome-wide significant association with one single-nucleotide polymorphism (SNP) [rs17612858, additive model: odds ratio, 2.19; 95% CI 1.66 to 2.90; *P* = 3.44 × 10^−8^], and genome-wide suggestive association with seven other SNPs (*P* < 1.0 × 10^−5^) in the HLA class II region of chromosome 6^16^. This finding was confirmed using a family-based association study involving 202 parent–child trios^16^.

The aims of this study were to undertake a second GWAS for podoconiosis in a much larger sample size to confirm this HLA association with higher confidence due to increased statistical power, and to determine whether other non-HLA loci were implicated. We included new samples from the Wolaita group and samples from two other ethnic groups, the Amhara and the Oromo, from other regions of Ethiopia where podoconiosis is endemic^17,18^. We then included the data from the original Wolaita cohort described above in the final analysis to maximise the power of the study. Through these studies we aimed to advance our understanding of the molecular processes involved in the development of a condition that clearly results from gene-environment interactions that could be relevant to other non-communicable diseases, especially those that are also associated with class II HLA genes. Further studies of the genetic basis of podoconiosis are also warranted to develop a point-of-care test to identify those at risk of developing podoconiosis and correctly diagnosis those with the condition. This would allow preventive measures to be taken to avoid the development of podoconiosis, for example through rational allocation of limited resources such as protective footwear, and help differentiate podoconiosis from other forms of tropical lymphoedema such as lymphatic filariasis.

## Results

### Study subjects, sample sizes and quality control measures

**Figure 1:**
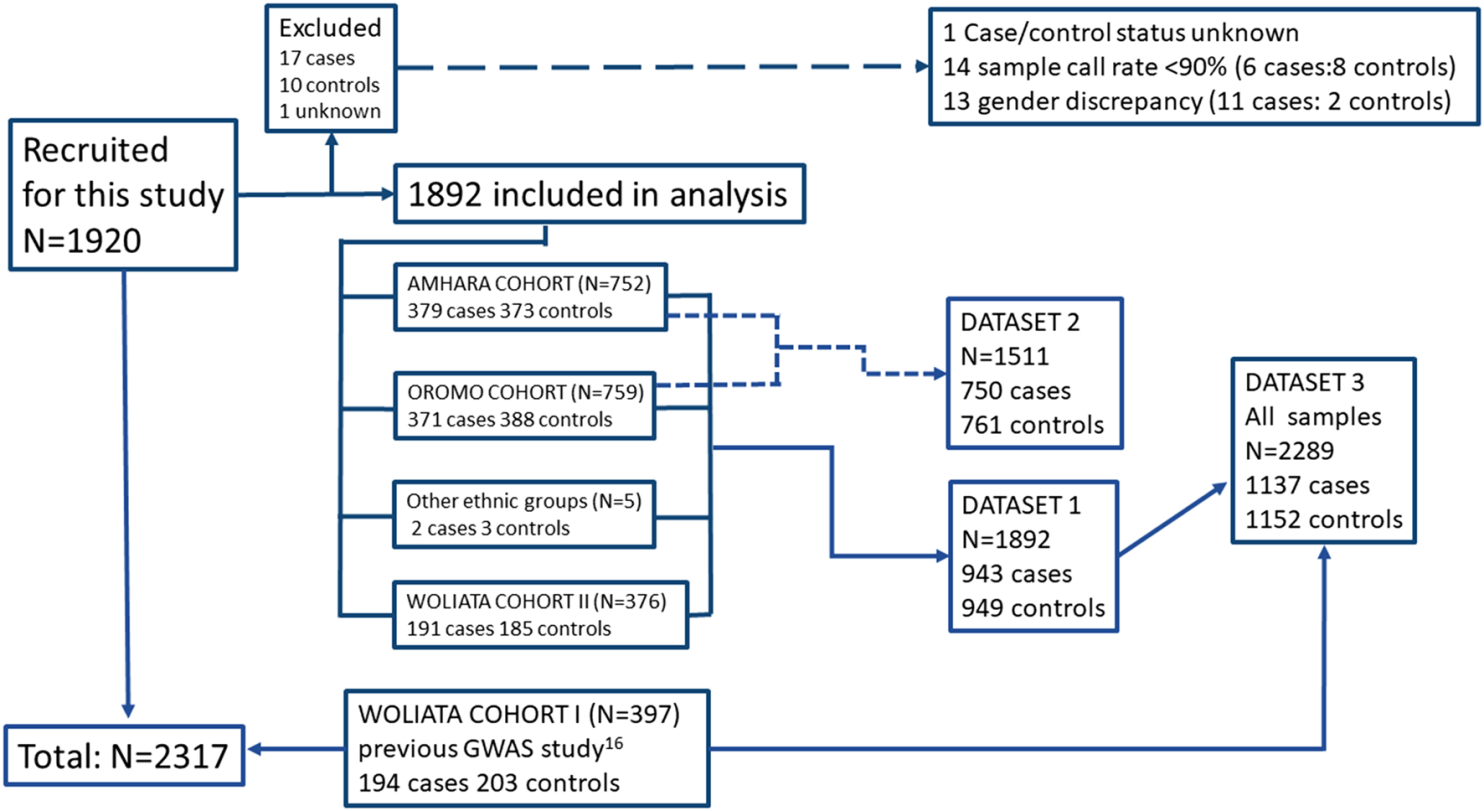
An overview of recruitment numbers and data analysis strategy. A total of 2317 DNA samples were available comprising 1920 new samples from three different Ethiopian ethnic groups (Amhara, Oromo and Wolaita) and 397 samples from a previous GWAS study on podoconiosis undertaken in a different cohort from the Wolaita group^16^. Twenty-eight new samples were excluded following QC checks. Data were analysed in three sets: dataset 1 comprised all newly recruited individuals from the three ethnic groups; dataset 2 comprised dataset 1 minus the Wolaita II cohort and ‘other ethnic groups’ samples; and dataset 3 comprised the total samples available for analysis from all cohorts.

DNA samples were available from a total of 2317 individuals. Of these, 1920 were newly-enrolled during this study from the Amhara, Oromo and Wolaita ethnic groups. The other 397 samples were collected from the Wolaita group during the first podoconiosis GWAS^16^ (194 cases and 203 controls). Figure 1 outlines the sources of the samples and the outcomes of the quality control (QC) process. The newly collected Wolaita cohort was designated Wolaita II and the previously collected cohort Wolaita I. Samples from 28 of the 1920 newly-enrolled individuals (17 cases, 10 controls and one unknown) were excluded during the QC process (see below). The remaining 1892 subjects comprised: 379 cases and 373 controls from the Amhara ethnic group; 371 cases and 388 controls from the Oromo ethnic group; 191 cases and 185 controls from the Wolaita group (Wolaita II); and 2 cases and 3 controls from other ethnic groups. Demographic and clinical information is summarised in Supplementary Table 1.

Details of sample QC, SNP QC and allele frequency information can be found in Supplementary Tables 2–4. After QC, a total of 2,210,858 autosomal SNPs were genotyped with an average genotyping call rate of 99%. Multidimensional scaling (MDS) analyses by identity by descent (IBD) with African and other 1000 Genome populations^19^ revealed clustering in one group, indicating that our samples were of similar ancestry (Supplementary Fig. 1). Quantile–quantile (Q-Q) plotting indicated a genomic inflation factor (λ) of 1.0406 confirming the absence of overt population stratification effects on the analysis (Supplementary Fig. 2).

Following genotyping and QC, data were organised into three datasets for analysis (Fig. 1). Dataset 1 comprised the data derived from all newly-enrolled individuals from all three ethnic groups (n = 1982), to conduct a second GWAS on podoconiosis in Ethiopia; dataset 2 excluded the Wolaita data in case any association identified in dataset 1 was driven by this group (in which the first GWAS found association) so data from the 1511 Amhara and Oromo only was analysed; and dataset 3 comprised data derived from all the samples collected for this study plus the samples from the first podoconiosis GWAS (Wolaita cohort I) that originally identified the HLA association with podoconiosis, to maximise the study power (n = 2289).

### Association results

**Figure 2:**
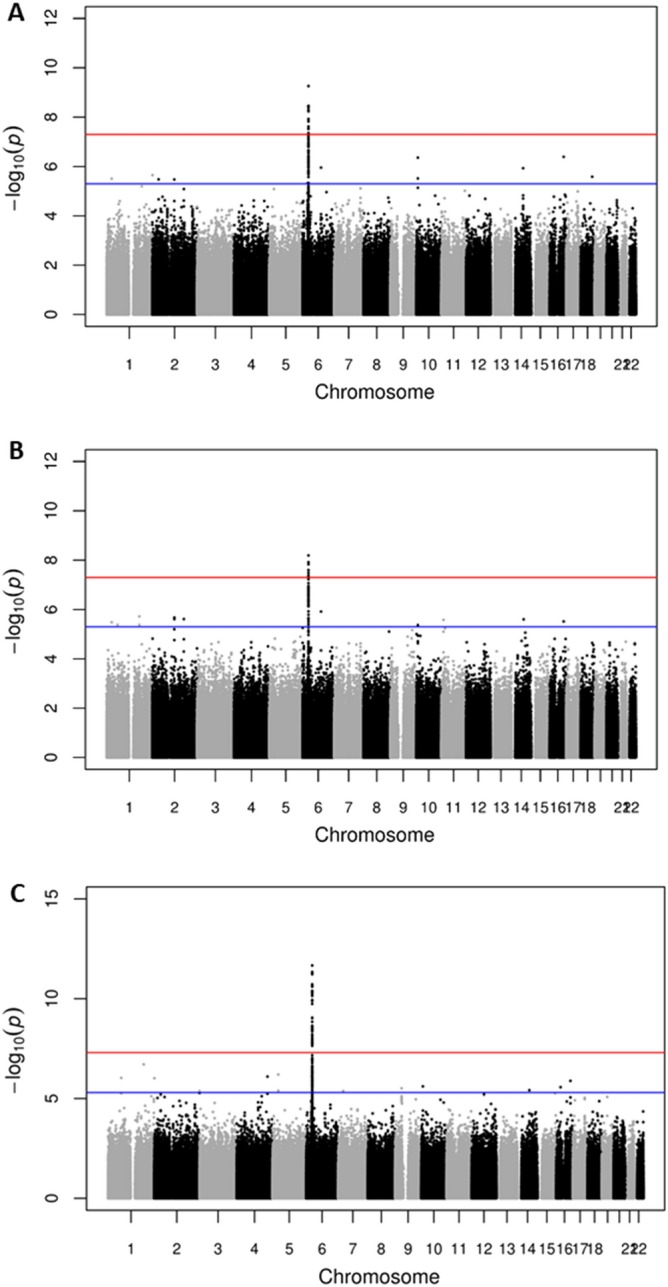
Manhattan plots showing genome-wide association with podoconiosis in Ethiopia. (**A**) dataset 1 comprising combined new samples from the Amhara, Oromo and Wolaita ethnic groups; (N = 1892, 943 cases and 949 controls); (**B**) dataset 2 from the Amhara and Oromo ethnic groups (N = 1511, 752 cases and 759 controls) and (**C**) dataset 3 comprising all available samples i.e. dataset 1 plus the samples from the previously-published podoconiosis GWAS undertaken in the Wolaita group (N = 2289,1137 cases and 1152 controls). For each plot, the x-axis indicates chromosomal position and the y-axis indicates the − log10 P value. Points between the blue and red lines shows suggestive association (*P* < 5.0 × 10^−7^) whereas points above the redline shows genome-wide significance (*P* < 5.0 × 10^−8^) results. The R programme was used to generate the plots^48^.

**Figure 3:**
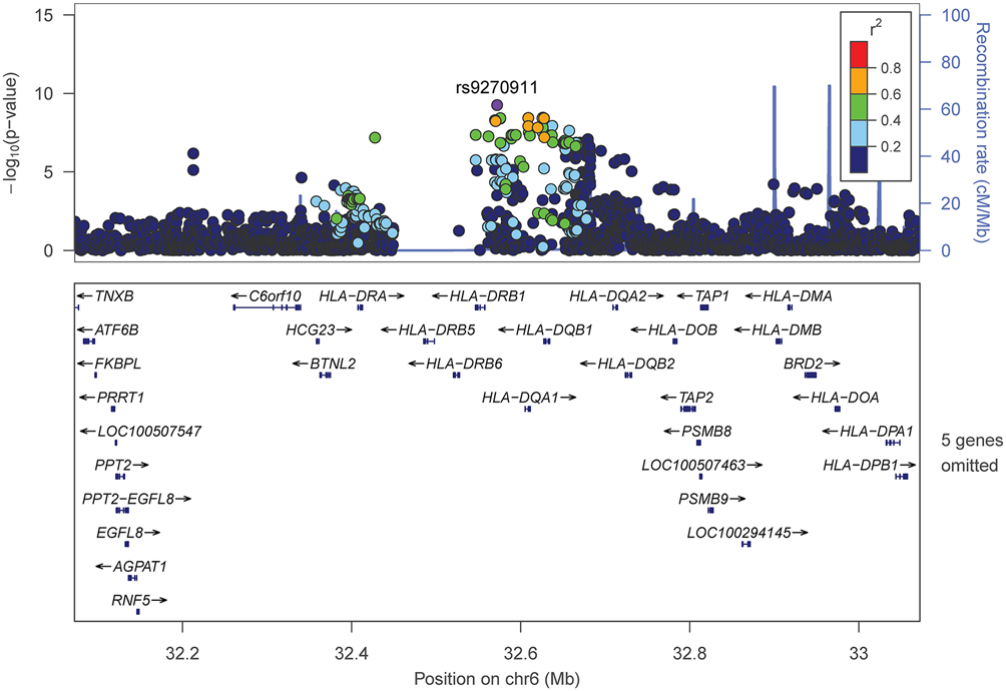
Association signals across a 1 Megabase region of the HLA class II region on chromosome 6 surrounding SNP rs9270911. Using LocusZoom, SNPs within 500 kb of the lead SNP rs9270911 were plotted based on their GWAS -log10 *P*-values, NCBI build 37 genomic position, and recombination rates calculated from the 1000 Genomes Project reference data. Each dot represents a SNP. The lead SNP rs9270911 is represented by a purple dot. The color scale of r^2^ values is used to label SNPs based on their degree of linkage disequilibrium with rs9270911. Most SNPS are dark blue indicating low LD (r^2^ < 0.2). The left y axis represented negative log10 *P* values, the right y-axis represents the recombination rate in centiMorgans (cM) per megabase (Mb). Genes found in the region are shown in relative position under the plot; arrows indicate the direction of transcription.

Focusing firstly on confirming the previously reported association between class II HLA variants and podoconiosis, analysis of the newly collected data (dataset 1 comprising 943 cases and 949 controls from all three ethnic groups) identified 14 SNPs that achieved genome-wide significance (*P* < 5.0 × 10^−8^) using an additive allelic model (Table 1). All of them were located in the HLA class II locus at 6p21.3 as shown in the Manhattan plot (Fig. 2A). The lead SNP was rs9270911 (*P* = 5.51 × 10^−10^; OR = 1.53; 95% CI = 1.34–1.74), a regulatory region variant closest to *HLA-DRB1* and located approximately 48 kb upstream of the previously reported index SNP, rs17612858. Using LocusZoom, SNPs within 500 kb of the lead SNP rs9270911 were plotted based on their GWAS − log10 *P*-values, NCBI build 37 genomic position, and recombination rates calculated from the 1000 Genomes Project reference data. (Fig. 3)^19^. To identify linkage-disequilibrium (LD)-independent SNPs, we utilised the clumping procedure in PLINK (using an r^2^ threshold of 0.1 and a window size of 250 kb) and identified one independent signal, rs9270911. LD-analysis of the significantly associated variants indicated that they were moderately correlated (Supplementary Fig. 3).

**Table 1:** Genome-wide significant results from a genome-wide association study (GWAS) on podoconiosis undertaken in three ethnic groups (Amhara, Oromo and Wolaita, dataset 1) from Ethiopia.

Chromo-some locus	SNP	Position (bp)*	A1	A2	Nearest gene	Ensembl annotation	MAF	*P*-value	OR (95% CI)	r^2^**
6p21.3	rs9270911^†‡^	32,572,202	T	C	*HLA-DRB1*	Regulatory region variant	0.4413	5.512E−10	1.53 (1.34 –1.74)	1
6p21.3	rs6906021^†^	32,626,311	C	T	*HLA-DQB1*	Downstream gene variant	0.4406	3.478E−09	1.50 (1.31 –1.72)	0.49
6p21.3	rs1129740	32,609,105	G	A	*HLA-DQA1*	Missense variant	0.4889	3.657E−09	0.67 (0.59–0.77)	0.76
6p21.3	rs482205^†‡^	32,576,009	G	T	*HLA-DRB1*	Intergenic variant	0.3621	3.754E−09	1.51 (1.32–1.73)	0.54
6p21.3	rs1063355^¥^	32,627,714	T	G	*HLA-DQB1*	3′UTR variant	0.4894	3.831E−09	0.67 (0.59–0.77)	0.76
6p21.3	rs9273349^¥^	32,625,869	T	C	*HLA-DQB1*	Downstream gene variant	0.4894	3.831E−09	0.67 (0.59–0.77)	0.76
6p21.3	rs643889	32,575,918	T	A	*HLA-DRB1*	Intergenic variant	0.3614	3.996E−09	1.51 (1.32–1.74)	0.53
6p21.3	rs477515	32,569,691	A	G	*HLA-DRB1*	Intergenic variant	0.3338	5.033E−09	1.51 (1.32–1.74)	0.64
6p21.3	rs2516049	32,570,400	C	T	*HLA-DRB1*	Intergenic variant	0.3343	5.847E−09	1.51 (1.32–1.74)	0.64
6p21.3	rs17205647	32,637,418	A	G	*HLA-DQB1*	Upstream gene variant	0.3721	1.177E−08	1.48 (1.30–1.69)	0.35
6p21.3	rs1071630	32,609,126	T	C	*HLA-DQA1*	Missense variant	0.4862	1.207E−08	0.68 (0.60–0.78)	0.75
6p21.3	rs6928482^†^	32,626,249	C	T	*HLA-DQB1*	Downstream gene variant	0.4447	1.482E−08	1.48 (1.29–1.69)	0.48
6p21.3	rs17843604^¥^	32,620,283	T	C	*HLADQA1*	Intergenic variant	0.4888	1.497E−08	1.47 (1.29–1.68)	0.8
6p21.3	rs4538748	32,657,505	C	T	*HLADQA1*	Intergenic variant	0.3743	2.377E−08	1.46 (1.28 –1.67)	0.34

SNP, single nucleotide polymorphism; A1, minor allele; A2, major allele; MAF, minor allele frequency; HWE, Hardy–Weinberg equilibrium.

*The position is based on the University of California Santa Cruz (UCSC) human genome build 19 (hg19/GRCCh37).

**r^2^:linkage disequilibrium (LD) with the lead SNP rs9270911.

^†^Independent SNPs (podoconiosis-associated SNPs that were independent of each other (r2 < 0.6) within 500 kb sliding windows, identified during annotation (see methods).

^‡^Lead SNPs: independent SNPs that are independent from each other (r^2^ < 0.1).

^¥^SNPs that showed suggestive genome-wide linkage with podoconiosis in the first GWAS^16^.

To investigate whether the HLA class II association identified in this new GWAS was driven by the Wolaita group—the population in which the HLA class II association was previously reported—we excluded the Woliata II data, analysing only the Amhara and Oromo data (dataset 2, 1511 samples, Fig. 1). We identified five HLA class II SNPs that reached genome-wide significance and the lead SNP was rs17205647 (*P* = 6.469E-09; OR = 1.56; 95% CI = 1.35–1.82; Fig. 2B and Supplementary Table 5). This variant is located upstream of *HLA-DQB1* and features in Table 1.

Finally, we undertook a genome-wide association analysis of all available data including those generated from the Wolaita I samples in which the HLA class II association was first reported (2289 samples, dataset 3, Fig. 1). A total of 47 SNPs reached genome wide-significance, and the lead SNP was rs9270911 (*P* = 2.25 × 10^−12^; Fig. 2C and Supplementary Table 6). Two SNPs (rs17205647 and rs4538748) were significantly associated in all three dataset analyses in this study. Three SNPs (rs9273349, rs1063355, and rs17843604) which showed suggestive association with podoconiosis in the original GWAS study (i.e. genome-wide significance P values of > 5.0 × 10^−8^ but < 1.0 × 10^−5^)^16^, showed genome-wide significance in this more highly-powered analysis. Analysis of the larger combined dataset (N = 2289) did not identify any new loci outside the HLA class II region.

Since the lead SNP in the original study (rs17612858) was not included in the Illumina HumanOmni2.5 array used in this study, we determined the pairwise LD value between our top SNP (rs9270911) and rs9273349 which was the next best-scoring SNP in the original study (*P* = 3.49 × 10^−07^) that was also represented on the Illumina HumanOmni2.5 array. The LD value between these two variants was r^2^ = 0.75 (D' = 0.996046), indicating that the SNPs are in high LD with each other.

### Functional mapping and annotation results

Functional mapping and annotation of the 14 genome-wide significant SNPs using FUnctional MApping (FUMA)^20^ identified four independent (r^2^ < 0.6) significant SNPs (rs9270911, rs482205, rs6928482, rs6906021), of which two (rs9270911, rs482205) were found to be lead SNPs (r^2^ < 0.1). All were located within the genomic locus 6:32564784-6: 32667548. *HLA-DRB1* co-localises to this region at position 6:32578769-6:589836.

**Figure 4:**
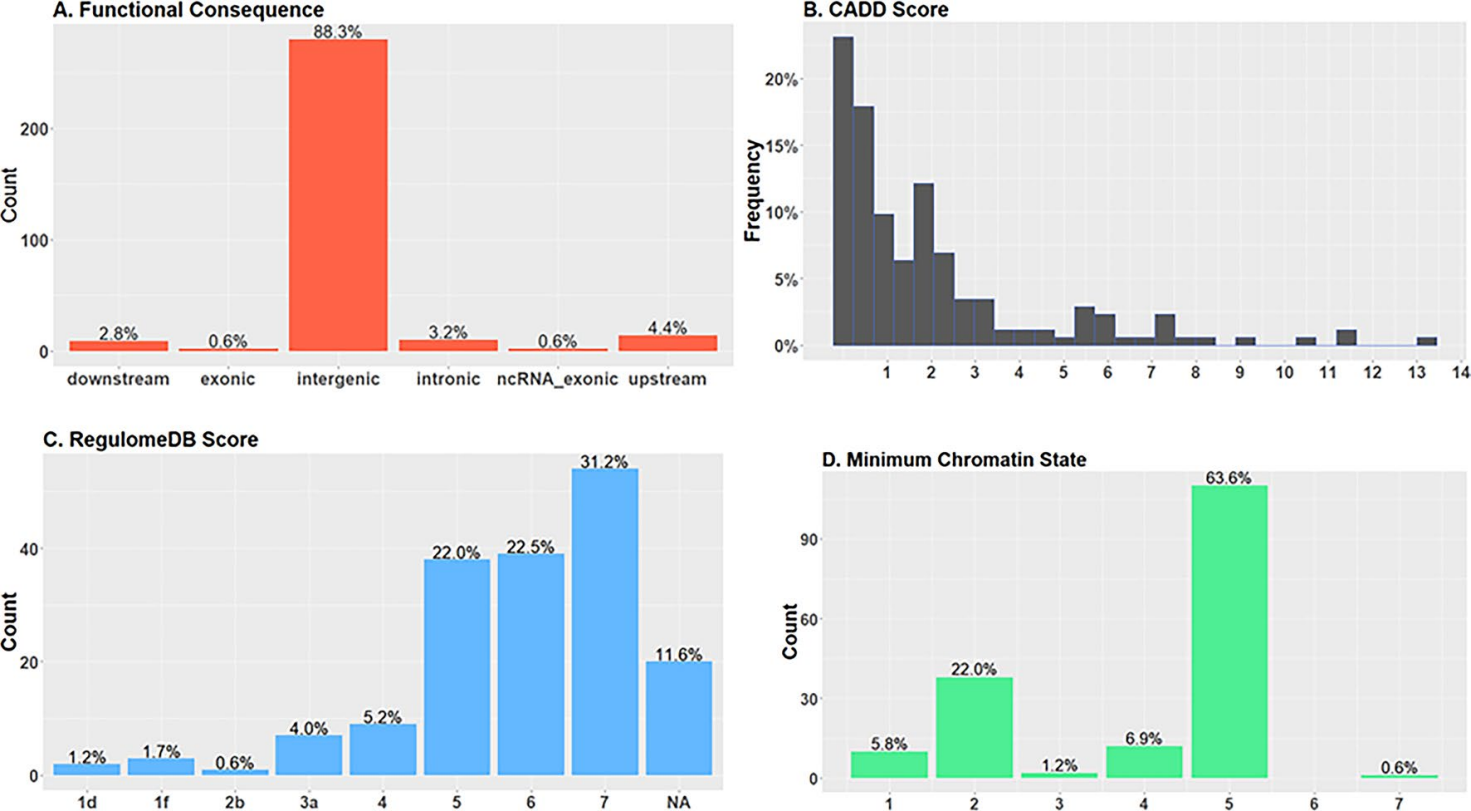
Functional annotation of 173 single nucleotide polymorphisms (SNPs) found to be in linkage disequilbrium (r^2^ >  = 0.6) with the four independent SNPs showing genome-wide significant association with podoconiosis. (**A**) Distribution of functional consequences of SNPs in genomic risk loci (**B**) Distribution of combined annotation dependent depletion (CADD) score for SNPs in genomic risk loci. A score greater than 12.37 indicates deleterious SNPs. (**C**) Distribution of RegulomeDB score for SNPs in genomic risk loci. A score less than 4 indicates regulatory function. (**D**) Minimum chromatin state for 127 tissue/cell types for SNPs in genomic risk loci. A score less than 7 indicates open chromatin state, indicating higher accessibility.

A total of 173 unique SNPs in this locus were in LD (r^2^ ≥ 0.6) with the four independent significant SNPs, and were functionally annotated. These SNPs were mostly located in intergenic regions (n = 140; 80.9%) (Fig. 4A). Except for one SNP (rs28366343), all SNPs had a combined annotation dependent depletion (CADD) score of less than 12.37, indicating that they are not deleterious (Fig. 4B). Twelve SNPs (6.8%) had a RegulomeDB score of less than 4 suggesting the SNPs have a regulatory role (Fig. 4C). Except for one, all SNPs (n = 172; 99.4%) were located in an open chromatin state as indicated by a minimum chromatin state less than 7 (Fig. 4D). Review of the 173 SNPs in LD with the four independent associated SNPs in the NHGRI-EBI GWAS catalogue^21^ revealed associations with other immune-mediated diseases such as inflammatory bowel disease, type 1 diabetes, multiple sclerosis, asthma, hayfever and allergy, and responses to Epstein-Barr virus and hepatitis B vaccine (Supplementary Table 7).

## Discussion

Using a genome-wide approach, we have confirmed the reported association between HLA class II variants and podoconiosis in an independent study that included 1892 new samples from three ethnic groups from Ethiopia, where podoconiosis is endemic. We found significant genome-wide association with 14 common variants in the HLA class II region on chromosome 6p21.3. The strongest association was with rs9270911, a regulatory variant located near *HLA-DRB1*. Other strongly associated variants were located near *HLA-DQA1* and *HLA-DQB1* which are contiguous and in LD with *HLA-DRB1*. The association was confirmed independently in the Oromia and Amhara groups, which have not previously been studied. The significance of the association was strengthened when the original Wolaita samples were included in the analysis (making a total of 2289 samples) with 47 common variants in the HLA class II region showing significant association with podoconiosis. This sample size was almost six times larger than that of the first podoconiosis GWAS (397 individuals) yet no variants outside the HLA class II region were found to be associated with podoconiosis suggesting a single major locus is predominantly responsible for genetic susceptibility to podoconiosis. This is consistent with a segregation analysis undertaken on multicase Wolaita families^15^, but even larger association studies would be required to identify loci with minor effects.

Genes within the HLA class II region encode numerous molecules that have critical functions in the adaptive immune system. The polymorphic *HLA-DRB1* encodes the β chain of the HLA class II glycoprotein HLA-DR, which together with the α chain (encoded by *HLA-DRA* which is not polymorphic) forms a functional antigen-binding heterodimer expressed on the surface of antigen presenting cells (APCs) such as macrophages, dendritic cells and B lymphocyte cells. Protein antigens processed by APCs into smaller peptides are loaded onto HLA molecules and presented to T-lymphocyte cells. Interaction between the HLA molecule, the peptide and the T-cell receptor (TCR) activates the T-cell to initiate an immune response specific to the antigen. Class II HLA molecules typically present peptide derived from exogenous (foreign) proteins to CD4^+^ T-cells whilst class I HLA molecules present endogenous (‘self’) peptides to CD8^+^ T cell. *HLA-DQA1* and *HLA-DQB1* encode α and β-chains for HLA-DQ molecules which have a similar role in antigen presentation to HLA-DR.

Association between HLA gene alleles and haplotypes and immune-mediated disorders is well established. These include autoimmune diseases such as type I diabetes mellitus (T1D), type 1 narcolepsy (T1N), coeliac disease and multiple sclerosis; infectious diseases such as leprosy and malaria; and aberrant reactions to molecules including beryllium and the anti-retroviral drug abacavir. In some cases there is a very strong predictable association with a single HLA allele—for example reaction to abacavir is associated with the HLA-B*5701 allele only and individuals can be genotyped for this variant before starting treatment^22^. In other disorders, such as multiple sclerosis, a number of haplotypes comprising different alleles are associated with susceptibility to disease. This may reflect epistatic interactions between the alleles, or selection of certain combinations of alleles that are more protective, through the ability to present a broader range of pathogen antigens than randomly inherited alleles would (bearing in mind the HLA has evolved to protect against pathogens)^23,24^. It is, therefore, important to extend our studies in podoconiosis from SNP associations to more informative HLA allele and haplotype associations towards identifying functionally important variants that underlie the molecular mechanisms of disease. Our previous work using direct HLA typing found association between the HLA-DRB1*0701, DQA1*0201 and DQB1*0202 alleles, and the HLA-DRB1*0701-DQB1*0202 haplotype and podoconiosis^16^. We also performed HLA imputation using SNPs to demonstrate that HLA class II alleles can be predicted from SNP genotype data with a high level of accuracy at intermediate (two-digit) resolution in an African population^25^. Further HLA typing and haplotype studies are being undertaken.

Despite the confounding effect of LD in the region, progress has been made towards understanding the mechanisms for some of these associations that will inform further studies as we seek to understand the molecular pathogenesis of podoconiosis. One of the fundamental postulates of autoimmunity is that the immune system mistakes self-antigens for foreign antigens to which the immune system has previously been exposed to and mounts a cross-reacting response to cause inflammation. Molecular mimicry is one mechanism by which this can happen, where the foreign antigen is derived from a microbe but closely resembles a self antigen^26^. The autoimmune disease T1N is a well-characterised example, where hypothalamic cells that secrete hypocretin (a neuropeptide that stimulates wakefulness) are destroyed by auto-reactive CD4^+^ T-cells. The disorder is associated with the HLA-DQ heterodimer DQ0602 comprising α- and β-chains encoded by HLA-DQA1*0102 and HLA-DQB1*0602 alleles respectively, with risk further increased for HLA-DQB1*0602 homozygotes^27^. The incidence of T1N cases increased after the 2009 H1N1 influenza pandemic and cases followed H1N1 influenza vaccination^28^. Screening of hypocretin and H1N1 influenza hemagglutinin identified a common peptide that could bind to HLA-DQ0602 and initiate T-cell mediated responses *in vitro*^29^. Whilst molecular mimicry involves peptides usually derived from microorganisms and there is no epidemiological evidence linking podoconiosis with infection, soil harbours numerous microbes many of which exist in a viable but non-culturable state and have yet to be characterised^30^, as well as other organic matter that could potentially be the source of a cross reactive peptide in podoconiosis.

Soil is also rich in metal ions and these can also trigger inflammatory disorders defined by their HLA associations. In chronic beryllium disease, granulomatous lung pathology develops in response to inhaled beryllium (an alkaline earth metal) in individuals carrying *HLA-DPB1* alleles that encode glutamic acid at position 69 of the HLA-DP β-chain. This allows positively-charged beryllium molecules to bind in the antigen-binding pocket to alter both the charge and conformation of the HLA-DP molecule allowing it to bind naturally-occurring peptides to create a ligand for pathogenic CD4^+^ T-cells^31^. Podoconiosis arises from prolonged contact with soil that is rich in metal ions. Alternatively, a soil mineral could induce changes in the structure or charge of a self-peptide or epitope, for example through post-translational modification, to create a neopeptide that is immunogenic, making this an attractive avenue to pursue in ongoing research on the soils that are involved in the aetiology of podoconiosis.

In many autoimmune diseases the self-peptide has yet to be identified, but progress has been made towards understanding the basis of the HLA association. Linkage between *HLA-DRB1* and T1D, in which an autoimmune process destroys the insulin-secreting β-islet cells of the pancreas, was well-established by family studies decades ago (and the role of HLA since confirmed by large-scale association studies^32^). Further studies at the time revealed a stronger association with *HLA-DQB1*^33^. Alleles that encoded the neutral amino acids serine, alanine or valine at position 57 of HLA-DQ1 were associated with susceptibility whilst aspartic acid at this position correlated with resistance to T1D. This finding was replicated in a Spanish population study, which also discovered that *HLA-DQA1* alleles encoding arginine at position 52 were associated with T1D^34^. These amino acids positions are located within the antigen-binding pockets of the HLA molecules suggesting amino acid substitutions influence molecular interactions within the pocket and alter the repertoire of peptides they present. Variations at other amino acid positions within the peptide binding pockets of both HLA-DQB1 and HLA-DRB1 molecules have also been shown to influence susceptibility and certain heterozygote HLA combinations appear to increase the risk of T1D in a synergistic manner^35^. Work is ongoing to identify the peptide that triggers immune response in diabetes—a number of candidates including autoantigens such as insulin are under investigation^36^.

Further elucidation of the HLA alleles that are associated with susceptibility or resistance to podoconiosis^16^ and amino acid sequence analysis are required to further understand the role of HLA in the pathogenesis of podoconiosis. A better understanding of the HLA molecular structure could also allow prediction of the antigen(s) and its epitopes, which are unknown for podoconiosis, in parallel with ongoing studies in soil from endemic areas. In addition to the scientific advances made through the study of the molecular pathogenesis of podoconiosis and its contribution towards understanding gene environment interactions in complex traits, there are practical public health implications of this molecular work that have a wider impact for affected communities and disease control globally. Limited resources such as protective footwear can already be targeted to families with a history of podoconiosis. Confirmation of the class II HLA association could further refine this approach to identify those at risk who do not have a family history. Careful engagement with communities would be required to avoid increasing stigma associated with podoconiosis^37^. Further effort may now be invested in developing a diagnostic test for podoconiosis, which is currently lacking. The condition affects up to 8% of people in affected communities^3^ and identification of a genetic marker, if not the causal variant, would refine identification of those at risk in whom podoconiosis could be prevented through consistent use of footwear. This would represent a major advance towards disease elimination.

There is a relative dearth of genetic data derived from African populations^38^ and this study has contributed detailed genotype data from three different ethnic groups to existing datasets. Modern humans evolved out of Africa and African populations are genetically more diverse than any other populations. Being older, population structure and linkage disequilibrium patterns are more complex and many of the tools used to study genetics in health and disease were developed in European-ancestry populations which do not fully capture this diversity^39,40^. This issue has been partly addressed through the availability of denser arrays that give better genome coverage, including the development of a 2.5 M African-specific GWAS array by the Human Health and Heredity in Africa (H3 Africa) Consortium^41^. However, there remains a need for population specific data to give better representation of the approximately 2000 ethnolinguistic groups that live in Africa^39^.

Detailed HLA type data are lacking for African populations despite the role of HLA in immunity to common fatal infectious diseases such as malaria, HIV and tuberculosis. Better understanding of HLA in this context is also relevant to the development of new or better vaccines for such diseases. A future application of our data will be to develop a tool to predict HLA types from selected genotype data once we have HLA typed our study cohort, obviating the need for the time-consuming and expensive methods currently required for HLA typing prior to organ transplantation.

In conclusion, this project replicates the finding that HLA class II gene loci have significant associations with podoconiosis. The involvement of immune mechanisms in podoconiosis susceptibility seems plausible^42^ and further research is required to identify causal variants and further characterise the molecular basis of the association.

## Methods

### Study population and datasets

This was a population-based case–control study involving individuals from three ethnic groups (Amhara, Oromo, and Wolaita) from four different zones of Ethiopia. The Amhara samples were collected from 12 *woredas* (districts) in the East Gojjam and West Gojjam Zones. The Oromo samples were collected from 6 *woredas* in the East Wellega Zone. The Wolaita samples comprised a newly collected cohort collected from 9 *woredas* within the Wolaita Zone (Wolaita II) and those from the original cohort (Woliata I) which are described in more detail elsewhere^16^.

Three genome-wide analyses were conducted (Fig. 1) using (a) the Amhara, Oromo and Wolaita II samples collected during this study aiming to confirm the results from the first GWAS and determine whether loci outside the HLA class II region could be identified (dataset 1), (b) the Amhara and Oromo samples, but not the Wolaita II samples, to evaluate whether any positive findings from dataset 1 were driven by the Wolaita data since this was the group in which the HLA association was first identified (dataset 2) and c) samples from all individuals recruited during this study combined with the Wolaita I samples to examine the strength of the association and to identify novel signals (dataset 3). Sociodemographic characteristics and clinical information were collected for newly enrolled participants.

### Case–control definition

Cases and controls were defined as described previously^16^. Briefly, cases were adults (aged 18 and above) with lymphoedema typical of podoconiosis and were resident in the study area for at least 5 years. Controls were healthy adults aged 50 and above who were resident in the podoconiosis endemic area for a minimum of 25 years and had no family history of podoconiosis and did not consistently use shoes. The age limit was chosen to allow sufficient contact with the volcanic soil over time for susceptible individuals to develop disease and not be mis-classified as controls^16^. Clinical assessment and disease staging were conducted by experienced nurses working at either the International Orthodox Christian Charities (IOCC) Debre Markos branch (Gojjam), the Catholic Church Clinic in Nekemte (Wellega), or Mossy Foot International in Wolaita using a validated clinical staging system for podoconiosis^43^. There is no proven diagnostic test for the disease.

### Power calculations

The online version of the GAS Power Calculator^44^ was used to calculate the power of the study using the followings assumptions in an additive model: significance level threshold of 5.0 × 10^−08^ and disease prevalence of 4%^4^. Different scenarios for the MAF and Odds Ratio (OR) were also used to estimate power for this study.

### Genotyping and quality control (QC)

DNA extracted from saliva samples using the Oragene saliva DNA kits (DNA Genotek, Ontario, Canada) was genotyped using the Illumina HumanOmni25-8 v.1–2 chip at the Wellcome Trust Sanger Institute (WTSI), Hinxton, UK. Standard quality control (QC) procedures^45^ were performed using PLINK v1.9^46^. Samples were excluded for cryptic relatedness/duplication (pi_hat > 0.09), gender discrepancy (based on the presence of heterozygous haploid genotypes), autosomal heterozygosity (> 3 SD from the mean), and call rate (< 90%). Outlier samples were also checked visually from multidimensional scaling (MDS) and principal component analysis (PCA) plots and were removed from downstream analysis. SNPs that deviate Hardy–Weinberg equilibrium (HWE), call rate (< 95%), and Minor Allele Frequency (MAF) (< 5%) were excluded. In addition, insertions and deletions (indels), copy number variants (CNVs), non-autosomal and non-biallelic SNPs were removed.

### Single-marker association analysis

Logistic regression under an additive model was conducted in PLINK adjusting for the first 10 principal components (PCs) as covariates. Age and shoe-wearing habit were not considered as covariates since they were controlled during participant recruitment. Genome-wide significance was set to *P* < 5.0 × 10^−8^ to account for multiple testing. Intensity cluster plots for the top associated SNPs were generated and manually examined to ensure only SNPs of high genotyping quality were considered for follow-up; poorly-called SNPs were removed from downstream analysis. Top associated SNPs showing good clustering were annotated using the Ensembl Variant Effect Predictor (VEP)^47^ (build GRCh37). Quantile–Quantile (Q–Q) and Manhattan plots were generated using R statistical software^48^. Regional association plots were made using LocusZoom^49^. The linkage disequilibrium (LD) structure for the genome-wide significant SNPs (*P* < 5.0 × 10^−8^) was plotted in Haploview v4.2^50^ using a haplotype definition described by Gabriel et al.^51^.

### Haplotype association testing

In order to determine the haplotypes driving our top associated SNPs, we conducted haplotype-based analysis. Briefly, SNPs spanning chromosome 6 region 28,889,000–33,055,000 bps were extracted using PLINK. Haplotype and missing genotype inference as well as haplotype association tests were carried out using Beagle v3.2.2^52,53^. The haplotype clusters which were found to be significantly associated with podoconiosis were further analyzed using the Beagle cluster2haps program to identify allele sequences that defined the haplotypes.

### Identification of genomic risk loci

We employed FUnctional MApping and annotation (FUMA), an online platform that combines various bioinformatics tools and data sources, on the summary statistics of the primary replication dataset to functionally annotate, map and prioritize the top associated variants^20^. First, genome-wide significant SNPs (*P* < 5.0 × 10^−8^) that were independent of each other (r^2^ < 0.6) within a 500 kb sliding window were identified (independent significant SNPs) using PLINK’s clumping procedure^46^. Lead SNPs were identified from the independent significant SNPs if they were independent from each other (r^2^ < 0.1). Candidate SNPs to be used for functional annotations and gene mapping were identified as all SNPs (either from the GWAS summary statistics or phase 3 1000G AFR reference population) that had a MAF of greater than 1% and were in LD (r^2^ ≥ 0.8) with at least one of the independent significant SNPs^19^. If the LD blocks for the independent significant SNPs were 250 kb up or downstream from the most up- or downstream SNPs from each LD block, they were merged as one genomic locus. Thus, each genomic locus could contain multiple independent significant SNPs and lead SNPs. SNPs that were previously reported to be associated with a phenotype in the NHGRI-EBI GWAS catalog^21^, and located in the same genomic loci were identified to provide further insight about the top associated variants.

### Functional annotation

Functional consequences of candidate SNPs were determined using the combined annotation dependent depletion (CADD) score^54^, RegulomeDB score^55^, and the 15-core chromatin state^56,57^ as implemented in FUMA. The CADD score estimates the deleteriousness of SNPs, whereas the latter two estimate regulatory functions. A CADD score greater or equal to 12.37 indicates a more deleterious variant, whereas a lower RegulomeDB score (on a scale of 1 to 7) indicates variants with a regulatory function. A 15-core chromatin state value of less than or equal to 7 (of 15 categorical states) is an indication of an open chromatin state (accessible genomic region).

### Ethical approval and informed consent

Ethical approval was obtained from the Armauer Hansen Research Institute (AHRI)/All Africa Leprosy and Tuberculosis Rehabilitation and Training Centre (ALERT) Ethics Review Committee (Ref:PO20/12) and the Ethiopian National Research Ethics Review Committee (Ref 310/577/06). Research Governance approval was given by Brighton and Sussex Medical School Research Governance and Ethics Committee (Ref 14/066/NEW). Written informed consent was obtained from each participant before enrolling them in the study. Rapid ethical appraisals was undertaken in all three study communities before sample collection began^58,59^. This qualitative approach used focus groups and in-depth interviews to explore the views and concerns of the community and other stakeholders regarding the study and research more generally. The findings allowed the design of a contextualized consent process whilst meeting international ethics standards for biomedical research involving human subjects.

## Supplementary Information

Supplementary Information.
