# Supplementary material for: Replication of HLA class II locus association with susceptibility to podoconiosis in three Ethiopian ethnic groups
Source: Sci Rep. 2021 Feb 8;11:3285. doi: 10.1038/s41598-021-81836-x (PMC7870958; doi:10.1038/s41598-021-81836-x)
Supplement: Supplementary file 1 — Supplementary Information. [file 41598_2021_81836_MOESM1_ESM.pdf]

Supplementary information

Replication of HLA class II locus association with susceptibility to podoconiosis in three Ethiopian ethnic groups

Tewodros Gebresilase[1,2], Chris Finan[3], Daniel Suveges[4], Tesfaye Sisay Tessema[2], Abraham Aseffa[1], Gail Davey[5], Konstantinos Hatzikotoulas[4], Eleftheria Zeggini[4], Melanie J. Newport[5], Fasil Tekola-Ayele[6]

Affiliations

1. *Armauer Hansen Research Institute (AHRI), Addis Ababa, Ethiopia*
2. *Unit of Health Biotechnology, Institute of Biotechnology, College of Natural and Computational Sciences, Addis Ababa University, Ethiopia*
3. Institute of Cardiovascular Science, Faculty of Population Health, University College London, London, U.K
4. *Wellcome Trust Sanger Institute, Hinxton, Cambridge, UK*
5. *Brighton and Sussex Centre for Global Health Research, Brighton and Sussex Medical School, Brighton, UK*
6. Epidemiology Branch, Division of Intramural Population Health Research, *Eunice Kennedy Shriver* National Institute of Child Health and Human Development, National Institutes of Health, Bethesda, MD, USA

Supplementary tables

Supplementary Table 1: Participant characteristics for subjects included in final data analysis

Characteristics	Category	Newly recruited study participants (Dataset 1, n=1892)		Previous Wolaita study cohort (n-397)		Dataset 1 and previous Wolaita cohorts combined (Dataset 3; n= 2289)	
		Cases (N=943)	Controls (N=949)	Cases (N=194)	Controls (N=203)	Cases (N=1137)	Controls (1152)
**Gender**	Male no (%)	433 (45.9)	535 (56.4)	93 (47.9)	87 (42.9)	526 (46.3)	622 (54)
	Female no (%)	510 (54.1)	414 (43.6)	101 (52.1)	116 (57.1)	611 (53.7)	530 (46)
**Age**	Mean (SD)	47.2 (14.2)	59.3 (8.2)	24 (6.8)	62.3 (10.8)	47. 4 (14.5)	59.7 (8.2)
	Median (range)	48 (18 - 95)	57 (50 - 99)	22 (18-50)	60 (50-102)	48 (18 - 90)	58 (50 - 95)
**Ethnicity**	Amhara	379 (40.2)	373 (39.3)			379 (32.6)	373 (32.3)
	Oromo	371 (39.3)	388 (40.9)			371 (33.3)	388 (33.7)
	Wolaita	191 (20.3)	185 (19.5)	190	203	381 (33.5)	388 (33.7)
	Hadiya	0	1 (0.1)	2	0	2 (0.2)	1 (0.1)
	Kenbata	2 (0.2)	1 (0.1)	1	0	3 (0.3)	1 (0.1)
	Gamo	0	0	1	0	1 (0.1)	0
	Unknown	0	1 (0.1)		0	0	1 (0.1)
**Clinical Stage*, left leg**	I	45 (4.8)	0	79 (40.7)	0	124 (10.9)	0
	II	522 (55.3)	0	61 (31.5)	0	583 (51.3)	0
	III	280 (29.7)	0	44 (22.7)	0	324 (28.5)	0
	IV	62 (6.6)	0	2 (1)	0	64 (5.6)	0
	V	34 (3.6)	0	8 (4.1)	0	42 (3.7)	0
**Clinical Stage, right leg**	I	51 (5.4)	0	81 (41.8)	0	132 (11.6)	0
	II	515 (54.6)	0	57 (29.4)	0	572 (50.3)	0
	III	284 (30.1)	0	49 (25.3)	0	333 (29.3)	0
	IV	58 (6.2)	0	1 (0.5)	0	59 (5.2)	0
	V	35 (3.7)	0	6 (3.1)	0	41 (3.6)	0

*Clinical staging was done using the podoconiosis clinical staging system as described by Tekola et al[1]

Supplementary Table 2: Summary of sample quality control (QC) data for datasets 1 and 3

Filter	Dataset 1 (n=1920)*		Dataset 3 (n=2339)*	
	**Number of failed samples**	**Remaining samples**	**Number of failed samples**	**Remaining samples**
Non-founder (siblings with parent (s))	0	1920	0	2339
Sample call rate (<90%)	14 (6 cases; 8 controls)	1906	21 (8 cases, 13 controls)	2318
Heterozygosity (>3 s.d. from the mean)	0	1906	30 (14 cases, 16 controls)	No deviate in PCA with 1000G
Sex check (F>0.8 male, F <0.02 female)	14 (11 cases; 2 controls; 1 unknown)	1892	14 (6 cases, 8 controls)	2304
Duplicates (>0.09)	0	1892	8 (2 cases, 6 controls)	2296
PCA/MDS clustering (visual inspection)	0	1892	5 (all controls)	2291
**Total**	**28** (17 cases, 10 controls, 1 unknown)	**1892** (943 cases, 949 controls)	**43** (16 cases, 27 controls)	**2291** (1147 cases, 1144 controls)

PCA, principal components analysis; MDS, multi-dimensional scaling.

*Dataset 1 comprised all study subjects recruited for this study from the three ethnic groups; dataset 3 also included Wolaita samples from the first podoconiosis GWAS

Supplementary Table 3: Summary of single nucleotide polymorphism (SNP) quality control (QC) result in the primary replication dataset 1 and combined replication dataset 3

Filter	dataset 1 (n=2330295)*		dataset 3 (n=2399785)*	
	# SNPs excluded	# SNPs retained	# SNPs excluded	# SNPs retained
Duplicate position (appear after merging the Wolaita I dataset and the rest of the samples (dataset 1))	0	2330295	12095	2387690
Fail strand Updating	0	2330295	8137	2379553
Non-biallelic/Copy Number Variant (CNVs)	0	2330295	0	2379553
Indels	0	2330295	0	2379553
Non-autosomes	55931	2274364	55627	2323926
Call rate (< 0.05)	58716	2215648	116531	2207395
Hardy-Weinberg Equilibrium (HWE) in controls (P < 10-4)	4790	2210858	5700	2201695
**Total**	119437	2210858	198090	2201695

*Dataset 1 comprised all study subjects recruited for this study from the three ethnic groups; dataset 3also included Wolaita samples from the first podoconiosis GWAS

Supplementary Table 4: Minor Allele Frequency (MAF) of the three datasets (see figure 1 main paper) after quality control (QC) procedure

Frequency	Dataset 1	Dataset 2	Dataset 3
**Monomorphic**	312868	325667	307397
**> 0 & <0.01**	250557	241758	561364
**>=0.01 & <0.05**	320923	321882	317227
**>=0.05**	1326511	1321552	1323105

Supplementary Table 5: SNPs that showed genome-wide significance with podoconiosis in dataset 2 (Amhara and Oromo samples)

Locus	SNP	Position (bp)	A1	A2	Gene	Ensembl Consequence	MAF	P-value	OR(95 % CI)	BONF
6	rs17205647	32637418	A	G	HLA-DQB1	upstream_gene_variant	0.371	6.469E-09	1.56 (1.35 - 1.82)	0.01187
6	rs6928482	32626249	C	T	HLA-DQB1	downstream_gene_variant	0.4378	1.207E-08	1.54 (1.33 - 1.79)	0.02215
6	rs4538748	32657505	C	T	-	intergenic_variant	0.3738	1.478E-08	1.54 (1.33 - 1.80)	0.02711
6	rs7744001	32626086	A	G	HLA-DQB1	downstream_gene_variant	0.3555	2.522E-08	1.55 (1.33 - 1.81)	0.04627
6	rs6906021	32626311	C	T	HLA-DQB1	downstream_gene_variant	0.4429	2.535E-08	1.54 (1.32 - 1.80)	0.04651

SNP, single nucleotide polymorphism; A1, minor allele; A2, major allele; MAF, minor allele frequency; OR, odds ratio; BONF, Bonferroni corrected P-value.

Supplementary Table 6: Top SNPs that showed genome-wide significance with podoconiosis in dataset 3 (all samples)

Chr	SNP	Position (bp)	A1	A2	Gene symbol	Ensembl consequence	MAF	P-value
6	rs9270911	32572202	T	C	-	regulatory_region_variant	0.44	2.25E-12
6	rs1063355	32627714	T	G	HLA-DQB1	3_prime_UTR_variant	0.49	5.54E-12
6	rs1129740	32609105	G	A	HLA-DQA1	missense_variant	0.49	6.54E-12
6	rs9273349	32625869	T	C	HLA-DQB1	downstream_gene_variant	0.49	7.18E-12
6	rs9273471	32628030	A	G	HLA-DQB1	splice_region_variant,intron_variant	0.38	1.95E-11
6	rs1071630	32609126	T	C	HLA-DQA1	missense_variant	0.49	1.98E-11
6	rs17843604	32620283	T	C	-	intergenic_variant	0.49	2.98E-11
6	rs482205	32576009	G	T	-	intergenic_variant	0.36	3.69E-11
6	rs9271498	32589282	A	G	-	intergenic_variant	0.3	3.88E-11
6	rs9271589	32591128	A	G	HLA-DQA1	upstream_gene_variant	0.3	3.88E-11
6	rs9271776	32594341	C	T	HLA-DQA1	upstream_gene_variant	0.3	3.88E-11
6	rs1130161	32610994	A	C	HLA-DQA1	3_prime_UTR_variant	0.3	4.06E-11
6	rs477515	32569691	A	G	-	intergenic_variant	0.33	4.19E-11
6	rs643889	32575918	T	A	-	intergenic_variant	0.36	5.30E-11
6	rs2516049	32570400	C	T	-	intergenic_variant	0.33	5.61E-11
6	rs9271488	32589000	T	G	-	intergenic_variant	0.3	1.07E-10
6	rs28366336	32561956	G	A	HLA-DRB1	upstream_gene_variant	0.29	1.17E-10
6	rs35445101	32546879	G	A	HLA-DRB1	missense_variant,splice_region_variant	0.29	1.67E-10
6	rs536810	32577497	T	C	-	regulatory_region_variant	0.43	8.08E-10
6	rs9274689	32636930	C	T	HLA-DQB1	upstream_gene_variant	0.43	9.35E-10
6	rs34061722	32599337	T	C	HLA-DQA1	intron_variant	0.34	1.58E-09
6	rs482044	32576064	C	G	-	intergenic_variant	0.4	1.68E-09
6	rs9268831	32427748	T	C	HLA-DRB9	non_coding_transcript_exon_variant	0.46	2.15E-09
6	rs3021058	32652359	C	A	-	intergenic_variant	0.43	2.40E-09
6	rs9274741	32637994	C	T	HLA-DQB1	upstream_gene_variant	0.44	2.93E-09
6	rs4642516	32657543	T	G	-	TF_binding_site_variant	0.43	3.02E-09
6	rs9275136	32650757	T	C	-	intergenic_variant	0.43	3.08E-09
6	rs9275141	32651117	G	T	-	intergenic_variant	0.43	3.08E-09

6	rs9275162	32652687	C	T	-	intergenic_variant	0.43	3.08E-09
6	rs9275169	32653721	A	T	-	regulatory_region_variant	0.44	3.21E-09
6	rs9275133	32650588	G	A	-	intergenic_variant	0.43	3.34E-09
6	rs2856695	32651894	G	A	-	intergenic_variant	0.43	3.50E-09
6	rs9275154	32652435	T	C	-	intergenic_variant	0.43	3.88E-09
6	rs2856693	32653283	T	C	-	intergenic_variant	0.43	4.35E-09
6	rs2856667	32665079	C	T	-	intergenic_variant	0.43	4.36E-09
6	rs17205647	32637418	A	G	HLA-DQB1	upstream_gene_variant	0.37	4.40E-09
6	rs73732611	32695075	C	T	HLA-DQB3	downstream_gene_variant	0.07	4.67E-09
6	rs28407322	32603355	C	T	HLA-DQA1	upstream_gene_variant	0.34	7.01E-09
6	rs4538748	32657505	C	T	-	intergenic_variant	0.38	7.74E-09
6	rs17427445	32663764	A	G	-	intergenic_variant	0.14	1.08E-08
6	rs35998847	32666997	A	G	-	intergenic_variant	0.14	1.08E-08
6	rs17427564	32667067	C	T	-	intergenic_variant	0.14	1.30E-08
6	rs10947332	32677440	A	G	MTCO3P1	upstream_gene_variant	0.14	1.40E-08
6	rs10484561	32665420	G	T	-	intergenic_variant	0.13	1.57E-08
6	rs12526612	32653598	A	G	-	regulatory_region_variant	0.14	1.69E-08
6	rs7744001	32626086	A	G	HLA-DQB1	downstream_gene_variant	0.35	1.83E-08
6	rs6689	32627700	G	A	HLA-DQB1	3_prime_UTR_variant	0.31	2.05E-08

Chr, chromosome; SNP, single nucleotide polymorphism; A1, minor allele; A2, major allele; MAF, minor allele frequency.

Supplementary Table 7: Traits associated with HLA SNPs in LD with podoconiosis-associated SNPs

SNP*	Chromosome	location (bp)	LD SNP**	PMID	Trait	Mapped gene	P-value
rs6928482	6	32627714	rs1063355	24837172	Ulcerative colitis	HLA-DQB1, HLA-DQB1-AS1	2E-6
rs482205	6	32570400	rs2516049	26819262	Epstein Barr virus nuclear antigen 1 IgG levels	HLA-DRB1 - LOC107986589	3E-20
rs482205	6	32570400	rs2516049	26819262	Epstein Barr virus nuclear antigen 1 IgG levels	HLA-DRB1 - LOC107986589	6E-14
rs482205	6	32570400	rs2516049	26819262	Epstein Barr virus nuclear antigen 1 IgG levels	HLA-DRB1 - LOC107986589	4E-9
rs6928482	6	32603487	rs3104367	27182965	Asthma	HLA-DRB1 - LOC107986589	1E-40
rs482205	6	32569691	rs477515	24282030	Hepatitis B vaccine response	HLA-DRB1 - LOC107986589	3E-19
rs482205	6	32569691	rs477515	23326239	Epstein Barr virus nuclear antigen immune response	HLA-DRB1 - LOC107986589	3E-13
rs482205	6	32569691	rs477515	18758464	Inflammatory bowel disease	HLA-DRB1 - LOC107986589	1E-8
rs6906021	6	32626311	rs6906021	23817569	Self-reported allergy	HLA-DQA1 - HLA-DQB1	7E-15
rs6906021	6	32626311	rs6906021	23817571	Allergic sensitization	HLA-DQA1 - HLA-DQB1	2E-12
rs6928482	6	32626130	rs7744020	24204295	Narcolepsy (age of onset)	HLA-DQA1 - HLA-DQB1	8E-9
rs6928482	6	32604372	rs9272346	17554300	Type 1 diabetes	HLA-DRB1 - LOC107986589	5E-134
rs6928482	6	32604372	rs9272346	18978792	Type 1 diabetes	HLA-DRB1 - LOC107986589	6E-129
rs6928482	6	32604372	rs9272346	23181788	Asthma	HLA-DRB1 - LOC107986589	2E-8
rs6928482	6	32626601	rs9273373	24388013	Asthma and hay fever	HLA-DQA1 - HLA-DQB1	4E-14
rs6928482	6	32677912	rs9275563	23472185	Multiple sclerosis (OCB status)	MTCO3P1 - LOC102725019	6E-11

SNP, single nucleotide polymorphism; bp, base pair; LD, linkage disequilibrium; PMID, PubMed identification; OCB, oligoclonal bands

*SNP significantly associated with podoconiosis in the GWAS reported in this paper (see table 1, main paper).

**Other HLA-located SNP in linkage disequilibrium (LD) with the SNP in column one shown to associated with the trait mentioned in column six and data published in PMID reference given in column five.

Supplementary Figures

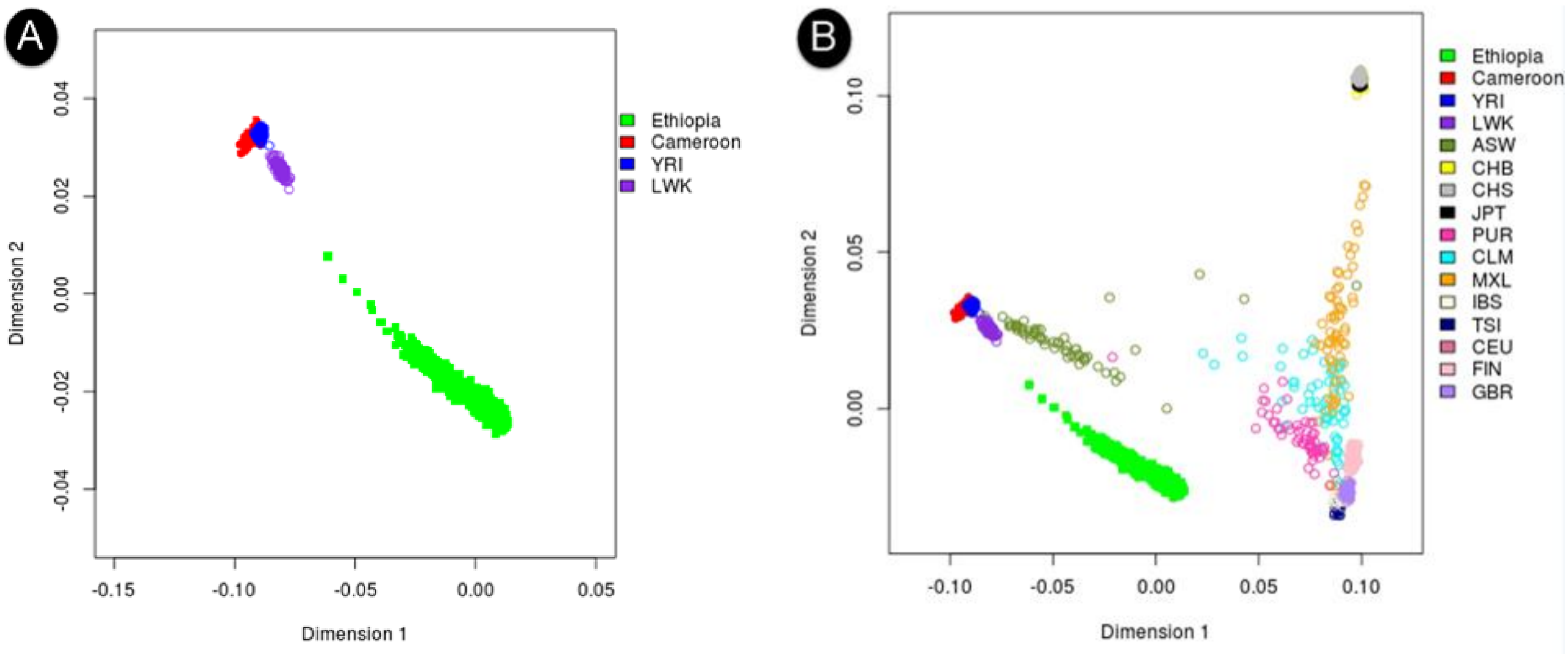

Supplementary figure 1: Multidimensional scaling (MDS) plot of the Ethiopian samples with (A) African (B) non-African populations included in the 1000 Genome project[2].

A

B

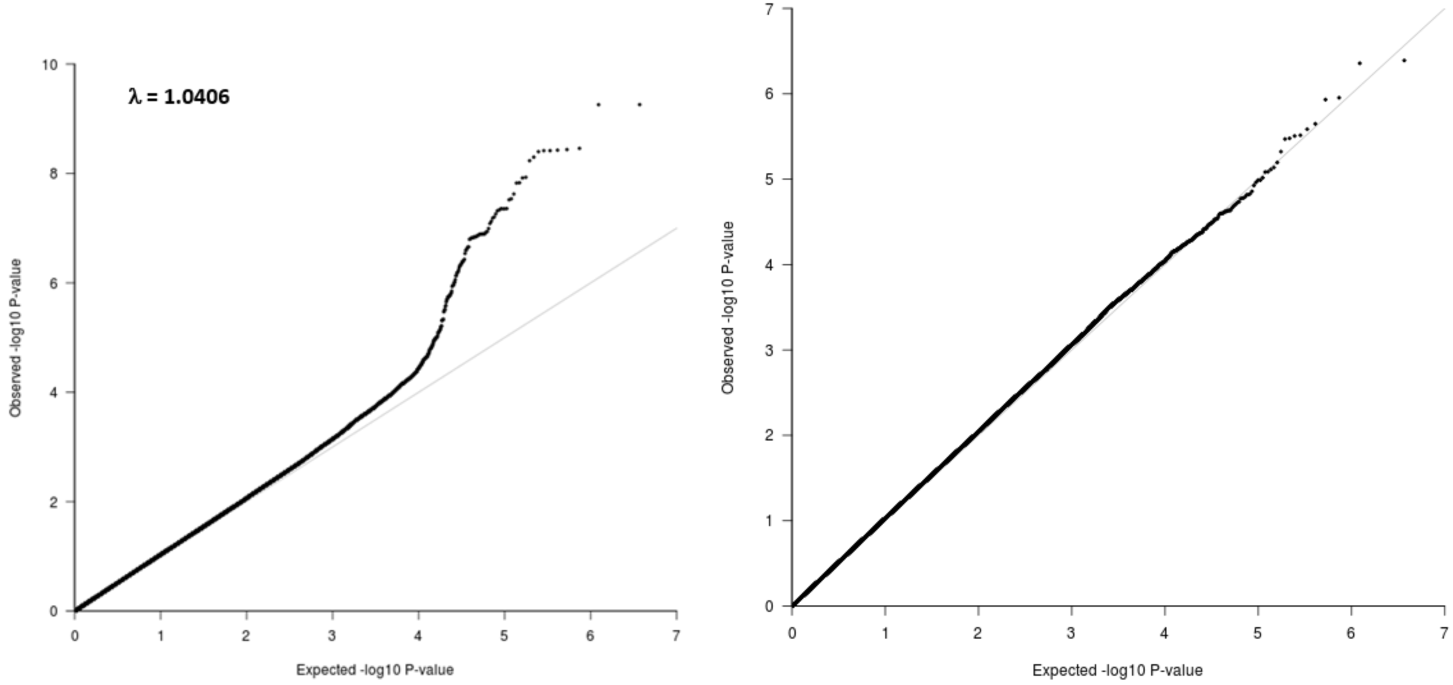

Supplementary figure 2. A) Quantile-Quantile (Q-Q) plot for dataset 1 which included 1892 samples collected from three different Ethiopian ethnic groups and B) after removal of SNPs from the HLA region (base pairs 29,000-33,000 on chromosome 6).

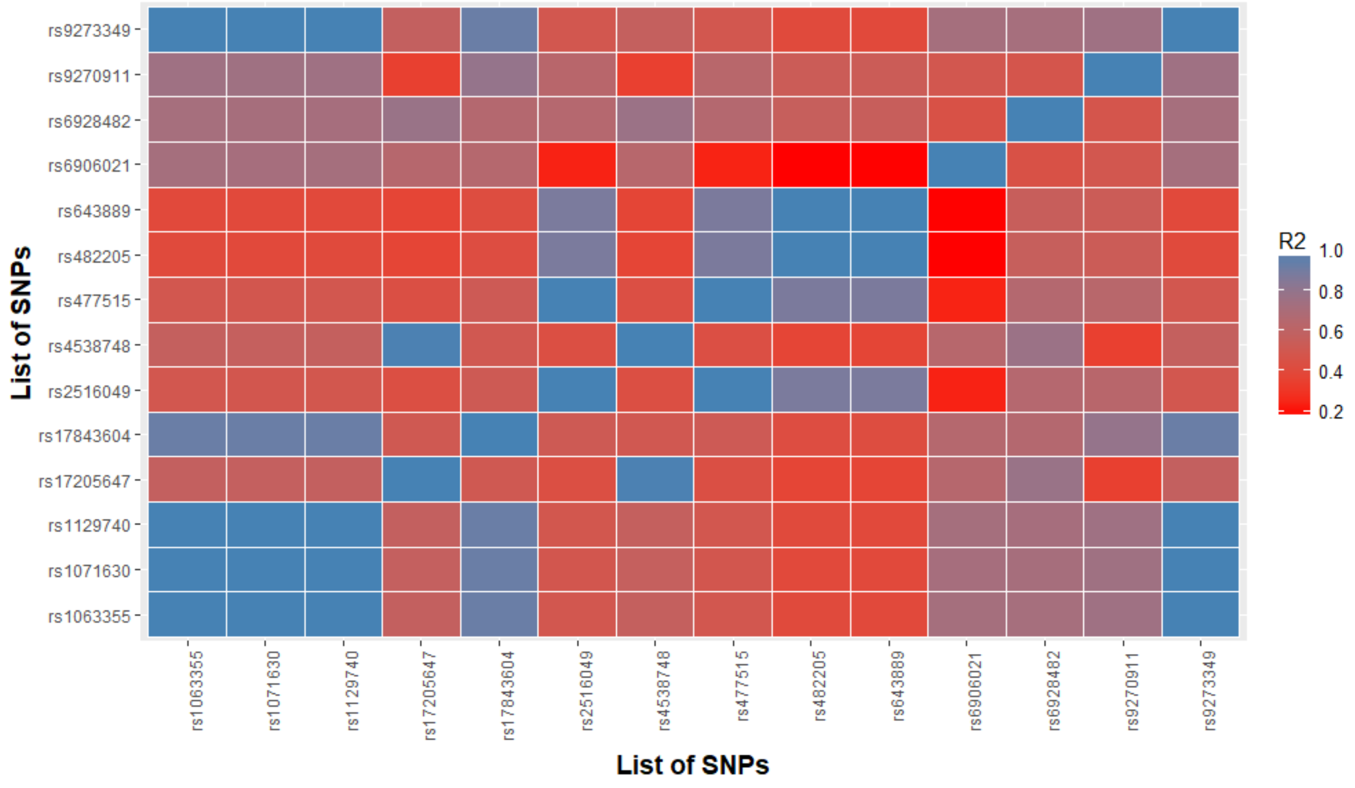

Supplementary figure 3: Heat map showing LD between the 14 associated (p < 5e-8) HLA class II SNPs on chromosome 6 from the GWAS of 1892 individuals from the three ethnic groups combined (Dataset 1).

References

1. Tekola, F., et al., *Development and testing of a de novo clinical staging system for podoconiosis (endemic non-filarial elephantiasis).* Trop. Med. Int. Health, 2008. **13**: p. 1277-83.

2. 1000 Genomes Project Consortium, et al., *A global reference for human genetic variation.* Nature, 2015. **526**(7571): p. 68-74.
